# Mr. Dawson, in Answer to Mr. Thomas

**Published:** 1807-03

**Authors:** G. P. Dawson

**Affiliations:** Sunderland


### *To the Editors of the Medical and Physical Journal.*

GENTLEMEN,

ZEALOUS friends are often more injurious to individuals than inveterate enemies. This reflection arose in my mind, mind, while I was reading Mr. Thomas's remarks on Dr. Bellamy's Case of Diseased Bladder. I am of opinion, his defence of his friend might have been dispensed with; I believe he would have served him better by preserving silence. Mr. Thomas acknowledges Dr. Bellamy's treatment of the case is open to objection, not consonant with his usual judgment, at one time improper, and that I have convicted him of error. It must be confessed, this is a very candid acknowledgement. But after it, why does Mr. Thomas seek to defend Dr. Bellamy? It is exceedingly singular. Is erroneous practice, and obvious error, defensible? No! But Mr. Thomas

——————— "displays a Christian spirit,
And tries to lift a lame dog over a stile.
Though not like Erskine, in the law a giant,
He must take up the cudgels for his client." Dr. Walcot.

I could have wished Mr. Thomas had pursued a different conduct. Dr. Bellamy has answered me in a manner so polite and gentlemanly, that I am inclined to behave to him with lenity, and to pay him all the respect he deserves. But in replying to Mr. Thomas, I must necessarily animadvert on Dr. Bellamy with apparent severity. I do it reluctantly, but what I owe myself, calls for it, and it is justified by the occasion. Considering my numerous observations on Dr. Bellamy's Memoir, it might have been expected, Mr. Thomas would have favoured me with a refutation of several; but on examining his letter attentively, I do not find he has attempted this in any instance but one, and that one was offered as a mere supposition.—Mr. Thomas has done me the honour of allowing my idea of the nature of the case to be correct, and is pleased to express his approbation of the treatment I have suggested.—The concessions which Mr. Thomas has so liberally made, render his defence of Dr. Bellamy, a supererogation of zeal. Permit me, Gentlemen, to reply to Mr. Thomas's remarks methodically. Mr. Thomas meaning not to dogmatize, but only to state an opinion, declares that my triumph is far from being complete; an opinion he hopes to substantiate by proof, pertinent and convincing. The singularity of this observation deserves notice. What does Mr. Thomas mean by triumph? I never sought any triumph, much less one which was complete. The love of truth impelled me to comment on Dr. Bellamy's Case; the desire of triumph I renounce. But had my object been been a triumph, it would be eminently successful. Can any critic enjoy a greater triumph, than that of demonstrating error and mal-practice? This I have done, Mr. Thomas has confessed it, and said his friend's treatment is open to objection, and contrary to his usual judgment. In the field of criticism, a complete triumph is never attainable, if by it Mr. Thomas means that a critic should expose many errors, and incontrovertibly prove his author has no claim, either to merit or compassion. No writer ever deserved universal censure. Mr. Thomas remarks, I describe Dr. Bellamy as perhaps, "experienced and well informed, but ignorant of the improvements of modern surgery." This is a quotation, not discoverable in my observations. It is a palpable perversion of my language, and makes me say what I never intended. In the last page, I have said Dr. Bellamy is no doubt experienced and well informed, and in a preceding line, that the treatment of his case was not agreeable to the rules of modern surgery. Surely, neither of these opinions can justify Mr. Thomas's quotation. To say Dr. Bellamy was experienced and well informed, was a compliment as handsome as it was sincere; and I appeal to every man, in whose mind veracity and candour reign, whether the assertion of Dr. Bellamy's treatment, not being agreeable to the rules of modern surgery, could mean that Dr. Bellamy was unacquainted with the various improvements in that art? To assert that it was irreconcilable to modern treatment, did not even in that instance, imply that Dr. Bellamy was ignorant of it, but merely that it had not been adopted. When Mr. Thomas writes again, I trust he will be more correct. In my writings, mis-quotation is studiously avoided. I am happy to hear Mr. Thomas's favourable representation of Dr. Bellamy's professional attainments, and that he has acquired experience at periods of distinguished glory to our country, and on "*various other*" occasions. Mr. Thomas admits the case was one of stricture, and contends, while no inflammatory symptom appeared, the practice at first adopted was perfectly justifiable. But I do maintain, that inflammatory symptoms, presented on the first day Mr. Sharp applied to Dr. Bellamy, and this I shall prove to the satisfaction of impartial judges, by narrating the symptoms, and those of inflammation. The symptoms of Mr. Sharp, on the first day he complained are, *tumour, painful* when touched, with much *internal distress*; a sense of *fulness* and *aching* when the parts are at rest, and still more more when urine or fæces are evacuated, and likewise *an affection of his general health!*

Such were Mr. Sharp's symptoms, and now what are these which mark inflammation? Why, *tumour, increased action of the vessels,* either of the *inflamed* part alone, or of the *whole* system, *tension, pain,* and great *irritability!* Let these symptoms be compared, they are a fair enumeration, and prejudice itself must allow there is a decided coincidence.

The perinæal swelling was first inflamed, the inflammation rapidly increased, and terminated in suppuration. All this is known to the world, yet Mr. Thomas asserts for the first two days, there were no inflammatory symptoms, although on the first day the swelling was attended with fulness, pain, internal distress, and before the expiration of four, the fluctuation of matter was evident! Mr. Thomas approves of the early part of Dr. Bellamy's practice? What was that practice? Warm fomentations, and five grains of quicksilver ointment, rubbed twice a day on a tumour which was tense, painful, and distressing! He says, it is a practice to which he has often resorted, and would again, under similar circumstances. I hope not. Let not Mr. Thomas's friendship bias his judgment. I ask him as a gentleman, whether the rubbing of five grains of mercurial ointment upon a tumour, exceedingly painful, is not injurious, and indicative that the man who does it, is entitled to commiseration? Mr. Thomas has said, in a tumour unattended with inflammation, he would resort to the practice of Dr. Bellamy. Would he employ warm fomentations? would he rub quicksilver ointment upon the surface of the tumour? would he use no more than five grains? I should answer all these interrogatories in the negative. Such is the practice to which Mr. Thomas assents, and declares he would adopt! I am as well convinced as Mr. Thomas can be, of the efficaciousness of mercury, in removing diseased deposits, though I cannot subscribe to his mode of using the remedy. Mr. Thomas's ideas of the action of mercury on diseased deposits, must indeed be very circumscribed, if he imagines it can act in any other way, than by exciting absorption through the medium of the system. True, mercury cures liver and other affections, but how? Not by rubbing mercurial ointment upon the affected part, but where the absorbents are very numerous, and can most speedily convey it into the circulation. It is too clear for imbecility imbecility to doubt, or presumption to deny, that mercury is beneficial to a diseased deposit, only by affecting the constitution. To rub mercurial ointment upon a diseased part, indicates ignorance of its action. It is ridiculous to suppose it can be productive of advantage, adequate to the method I have mentioned. The best absorbing surfaces should always be selected, hence the abdomen, inside of the thighs and arms, are recommended. But the plan of applying mercurial ointment to a tumour, is more than ignorant; it is decidedly reprehensible and prejudicial. It irritates the surface, occasions inflammation, and induces suppuration in a tumour, which might be absorbed, but for its pernicious application. Mr. Thomas denies that the termination of Mr. Sharp's swelling proved it to be no chronic enlargement, as I confidently assert. I still adhere to that assertion, and it is confirmed by facts.

Mr. Thomas says, that thinking it not sufficient to condemn the whole medical treatment, I labour also to fix on Dr. Bellamy a charge, which, if proved, would reflect greatly on his prudence and skill as an operator. He alludes to my having supposed Dr. Bellamy had forced his catheter through Mr. Sharp's urethra, into an abscess in the perinæum. Mr. Thomas is mistaken, I do not labour at all; but he, as your readers will observe, labours with more assiduity, but certainly with less success, to make wrong appear right. I solemnly deny labouring to fix such a charge on Dr. Bellamy. I simply stated the supposition, which was warranted by the premises. For my justification, I beg leave to transcribe my language on the occasion. "Previous to the 30th, Dr. Bellamy has said, no urine passed by the abscess; but on this day, he informs us, about half an ounce was evacuated. He consoles himself, by observing, it was 'a reasonable event to expect, there evidently existing narrowness of the canal, passing the catheter requiring *considerable* trouble. *The obstructions may be felt breaking* down under it.' Was not an opening made through the urethra into the abscess by the point of the catheter. Illiberality or invidiousness I disclaim, but truth demands the question.—If I wrong Dr. Bellamy by the supposition, I am sorry for it; but if he wished to have prevented it, he should have been more guarded in his language." This extract is the best answer to Mr. Thomas's allegation. Mr. Thomas attempts to prove this supposition to be erroneous. For the sake of Dr. Bellamy, I hope it is; it is possible: but be that as it may, may, I shall proceed to defend it, and shew it stands unshaken by Mr. Thomas's confident refutation.

Whenever this accident happens, Mr. Thomas presumes it is the immediate, and not the remote consequence of violence offered to the urethra; that it shews itself the moment the catheter is thrust through the canal, or the first time the patient attempts to make water; but is never brought on gradually from a sloughing induced by such violence. To this assumption I cannot accede. That it is true in some cases, is unquestionable; that it is false in others is indisputable. If Mr. Thomas's position be correct, he declares my surmise falls to the ground. But whether it be correct, or incorrect, he will derive from it no advantage whatever. Mr. Thomas has referred to Dr. Bellamy's daily reports to controvert my surmise; I shall do the same to confirm it, and I firmly believe I shall succeed better than Mr. Thomas has done.

On the 27th of February, Dr. Bellamy, notwithstanding obstruction about the curve of the urethra, passed his catheter tolerably easy into the bladder. This proves its introduction was not uncommonly difficult. On the 28th, the catheter was again passed, but with more difficulty; much b lood came through it, from evident obstructions at the curve of the urethra, with several attempts, and some force. Strange indeed! Now let me claim attention:—On the first day the catheter was easily passed, and it could not be felt by the minutest probing through the wound. This demonstrates, that no connection then existed between the urinary canal and the abscess, and as the catheter was easy of introduction, it is fair to conclude it would be equally so the succeeding day, nay more so, if we are to credit Dr. Bellamy, who says, in his answer to me, the parts would be relaxed by suppuration. But on the second or succeeding day, the passing of the catheter required several attempts, considerable difficulty, some force, and brought away much blood! From whence came the *blood?* Dr. Bellamy shall answer the question:—"*from evident obstructions at the curve of the urethra !!!*" What is the conclusion? On the first day Dr. Bellamy was so fortunate as to find the constricted aperture, on the second he missed it, forced his catheter through the obstructions, and by it, induced a copious discharge of blood, according to his own confession. What could be the probable result? Immediate inflammation, and consequent sloughing! The event seems to verify the assertion.

tion. It is not my surmise, but Mr. Thomas's position which now falls to the ground! But admitting his position to be correct, it is readily refutable.

Mr. Thomas will remember, that on the 30th, when Dr. Bellamy discovered the injury, only half an ounce of urine was evacuated, and came from about the bulb of the urethra, the identical place where the point of the catheter encountered obstruction. Now it is exceedingly likely, the urine might pass into the abscess on the 28th, or second day, for half an ounce might be so mixed with the matter, and the cataplasm which was daily applied, as to elude either detection or suspicion. The probability of this is undeniable. Recollect Dr. Bellamy did not see Mr. Sharp make water, for on the 30th, he desired him to keep it until the evening dressing. It is true, Mr. Sharp told Dr. Bellamy, no urine passed by the abscess, but how could he know when it was always covered by poultices? and he could not ascertain the fact by sensation; as it is said on the 30th, the urine which passed, gave him no pain! In whatever way Mr. Thomas's position is taken, it is satisfactorily answered. Where now is Mr. Thomas's boasted refutation? Has my surmise by his means fallen to the ground? Would to God it were! but let me be spared the mortification of thinking it was accomplished by what he has advanced. I have strengthened it by damning proof; for him it stands like the monument. Is not the introduction of a catheter, a delicate operation, demanding care and tenderness? What says Mr. Hey of Leeds? "Whatever method of performing this operation is pursued, the catheter should be introduced with the *greatest gentleness.* When any *obstruction* occurs, the design of the surgeon should be to *evade*, rather than *overcome* it. Unsuccessful *attempts* may render a case *extremely difficult*, which was not so before. I wish to impress upon the mind of my reader, that a *moderate force*, improperly directed, is capable of *injuring the urethra* in such a manner, as to render the operation almost (and without a just knowledge of the injury altogether) impracticable. It must be obvious to every surgeon, that *long continued* or *violent attempts* have a tendency to increase the *inflammation of the urethra.*" Such is the language of Mr. Hey in cases of retention of urine, where the catheter must be introduced. How poignantly does it apply to Dr. Bellamy, when it is beyond dispute, that there was no necessity for him to employ that instrument?

Permit

Permit me to ask Mr. Thomas, if the man who acknowledged to the world, that in introducing a catheter, he employed *force, considerable trouble, several attempts,* and *broke down obstructions in the urethra by its point, so as to produce a discharge of blood,* did not merit a *supposition* severer than mine? No one can be more sensible than I am, of the invidious task I have been executing. To sit down calmly, to convict a gentleman of having committed an injury which is reprehensible, where no provocation has been given, and no good can be derived, is an action which a generous mind will never contemplate with satisfaction. It is with me a matter of regret, that it has fallen to my lot. But it was imposed on me by well meant friendship; and conscious of the purity of my motives, I have not shrunk from its performance.

Mr. Thomas thinks himself justified, in assuming that my perspicacity, which would lead me to look beyond the ken of vulgar minds, has, in the point last discussed, hurried me away from the boundaries of sober consideration, into the regions of rash and premature conjecture, as your readers must recollect it did in another instance. Mr. Thomas means a singular Case of Delivery, which one of your Journals contains. I thank him for affording me an opportunity of saying a few words on the subject. On reading the case, I was struck with a degree of scepticism, which was corroborated by the omission of particulars, relative to sexual intercourse, and other important circumstances. The veracity of the gentleman concerned in the case, I did not dare to doubt; but all men being prone to error, it was possible they might be mistaken. Nothing can solve a doubtful question so well, as liberal investigation. Certain of this truth, I ventured to express my sentiments with becoming modesty; and to guard against the shadow of presumption, I purposely named them conjectures. The event answered my expectations. The reporter replied to them; he thought them handsome, and the case became more lucid, and better substantiated. But aware of the ambiguity of his narrative, Mr. Cribb honoured me with a private letter, containing facts, unfit to meet the public eye, and assured me, he was gratified by my remarks, as they enabled him to clear up, what others, as well as myself, might consider obscurities in his first paper. Dr. Crane of Boston, endeavoured to explain the case, and though he believed my conjectures false, said they were highly ingenious. To the politeness of Mr. Cribb and Dr. Crane, I feel myself much indebted.—

But the conjectures which Mr. Cribb pronounced handsome, and Dr. Crane ingenious, are by Mr. Thomas asserted to be rash, premature, and beyond the boundaries of sober consideration. This is an assertion, that can neither be defended on the score of argument, nor justified on that of manners. To use Mr. Thomas's own language, it is an observation, not at all relevant to the question under discussion, and certainly not worthy of that liberality of sentiment, which I am bound to suppose he possesses. In what way did I hurry myself beyond the boundaries of sober consideration? Why were my conjectures rash? I demand to know why they were premature? Are those conjectures rash, whose avowed object is the elucidation of obscurity? Are remarks premature, or culpable, when they are offered with candour and modesty; when they seek to develope mystery, and when they effect the purpose for which they are intended? Were my conjectures candid and modest? Enquire of Mr. Cribb. Did my conjectures throw additional light on a case at once curious, interesting, and mysterious? Yes! If this is doubted, I refer the sceptic to the letters of Dr. Crane and Mr. Cooper. With what degree of justice then, can Mr. Thomas presume to say, my observations on Mr. Cribb's case were rash and premature? or by what right does he accuse me of hurrying myself beyond the boundaries of sober consideration? Mr. Thomas is certain your readers must recollect my conjectures; but afraid they should not, he, in a foot note, supplies them with exact references to all the papers which were written. It is impossible not to admire Mr. Thomas's kindness and attention. Mr. Thomas concludes by declaring, that in pointing out the difference between us, he has studiously avoided the very semblance of offence. To comment on this declaration, would be superfluous. Presuming on your indulgence, Gentlemen, I request to occupy a few pages of your Journal, in replying to Dr. Bellamy's answer. The importance of the subject, the information which it embraces, the remarks I have to offer, and the authorities I shall cite, embolden me to make this requisition. Stricture, abscess, and fistula, are no trivial topics; they deserve all the industry and acuteness of which intellect is capable.

I am, &c.

G. P. DAWSON.

*Sunderland, Jan.* 8, 1807.

